# Cell chirality in cardiovascular development and disease

**DOI:** 10.1063/5.0014424

**Published:** 2020-08-25

**Authors:** Tasnif Rahman, Haokang Zhang, Jie Fan, Leo Q. Wan

**Affiliations:** 1 Department of Biomedical Engineering, Rensselaer Polytechnic Institute, Troy, New York 12180, USA; 2 Center for Biotechnology and Interdisciplinary Studies, Rensselaer Polytechnic Institute, Troy, New York 12180, USA; 3 Center for Modeling, Simulation, and Imaging in Medicine, Rensselaer Polytechnic Institute, Troy, New York 12180, USA; 4 Department of Biological Sciences, Rensselaer Polytechnic Institute, Troy, New York 12180, USA

## Abstract

The cardiovascular system demonstrates left-right (LR) asymmetry: most notably, the LR asymmetric looping of the bilaterally symmetric linear heart tube. Similarly, the orientation of the aortic arch is asymmetric as well. Perturbations to the asymmetry have been associated with several congenital heart malformations and vascular disorders. The source of the asymmetry, however, is not clear. Cell chirality, a recently discovered and intrinsic LR asymmetric cellular morphological property, has been implicated in the heart looping and vascular barrier function. In this paper, we summarize recent advances in the field of cell chirality and describe various approaches developed for studying cell chirality at multi- and single-cell levels. We also examine research progress in asymmetric cardiovascular development and associated malformations. Finally, we review evidence connecting cell chirality to cardiac looping and vascular permeability and provide thoughts on future research directions for cell chirality in the context of cardiovascular development and disease.

## INTRODUCTION

Asymmetry is everywhere in nature: from the coiling of snail shells, the winding of climbing plants, to the arrangement of seeds and petals on sunflowers. They all exhibit an inherent asymmetry or chirality. This deviation from symmetry is less evident in the case of humans, and indeed most animals, who demonstrate apparent bilateral or radial symmetry externally but have stark asymmetries internally. The perfect examples are sea urchins, which look radially symmetrical but show Left-Right (LR) asymmetry during their larval stages—determining from which side of the larva the eventual organism is derived.^1^ Humans, like other bilaterally symmetric animals, look similarly symmetric from the outside, but our visceral organs are asymmetric: particularly the unpaired organs like the heart, stomach, intestines, and liver. The asymmetry of the cardiovascular system, due to its crucial function and intriguing morphology, has been particularly well studied.

The heart is the first organ to form during embryogenesis, immediately following the primitive streak formation and, consequently, also represents the first physical evidence of LR symmetry breaking in the bilateral embryo. The heart starts as an LR symmetric tubular tissue referred to as the primordial heart tube (HT).^2–5^ The HT forms from a bilateral collection of cardiac progenitor cells (CPCs) within the lateral plate mesoderm (LPM) known as the anterior (or first) and posterior (or second) heart fields (FHF and SHF, respectively), which migrate to the midline of the embryo and form the linear HT.^4,6^ The initial symmetry breaking occurs during the process known as C-looping, during which the linear heart tube bends ventrally and twists dextrally, forming a right-handed loop, referred to as the cardiac c-loop (Fig. 2).^5^ The looping direction is chiral by nature, such that the apex of the subsequent ventricular chambers points to the right side of the body, while the heart itself is positioned on the left side [Fig. 2(b), left panel].^3,7^ Any deviance in the normal positioning of the heart may lead to severe congenital heart diseases, which are often fatal.^7,8^

Besides the critical “pump,” heart, the main portion of the cardiovascular system, is the massive network of blood vessels spreading throughout the body, which fuels all the organs and tissues with oxygen and other necessary nutrients while removing waste. The systemic circuit of vessels allows blood to flow out from the heart through the aorta and flow back from the rest of the body, and the pulmonary circuit bridges blood flow between lungs and the heart. Although symmetrical branching is seen in capillary vessels, the vessel trees on larger scales are predominantly asymmetrical.^9^ The aorta and pulmonary arteries, as the major arteries divided by the aorticopulmonary septum, are essential traces of vascular asymmetry after the dextral C-looping of the HT. Malformations in their laterality usually result in severe congenital defects.

Understanding the cellular, molecular, and biophysical mechanisms that regulate the development of cardiovascular LR asymmetry is of importance from both developmental and clinical perspectives. Much work has been done to elucidate the biophysical and cellular bases for the development of cardiovascular asymmetry in recent years, challenging several historically held perspectives while introducing and supporting novel hypotheses. Of particular interest is the recently discovered, intrinsic LR asymmetric property of eukaryotic cells: cell chirality. While the specific mechanisms of cell chirality development have been elusive, its manifestation has been observed both *in vitro* and *in vivo.*^10,11^ Cell chirality is often described as an LR asymmetric migration, alignment, or rotation of cells, as well as the LR biased positioning and rotation of intracellular organelles and cytoskeletal proteins.^10,12–16^ Novel markers of cell chirality are still actively discovered, due to the ubiquitous nature of this property. Recent evidence from *in vitro* and *in vivo* experiments have suggested that this cell-level chirality may be responsible for determining the bulk LR asymmetries observed in the body plans and specific tissues of biological organisms.^17,18^ Like LR asymmetry of the body, cell chirality is also highly evolutionarily conserved, having been observed in human, avian, murine, piscine, and Drosophila cells.^10,15,19–21^ Significant work has also been done in recent years to further associate cell chirality to tissue-level asymmetry.^10,14,17–20^

In this report, we discuss recent progress in the field of cell chirality as it relates to cardiac c-looping and vascular asymmetry and explore the role of cell chirality in cardiovascular development and diseases.

## HEART LATERALITY

Morphological heart defects make up the largest portion of all congenital deformities in humans: almost half of all birth defects observed or about 0.8%–1% of all live births.^22^ Diseases of heart laterality, however, are much rarer. The two most common heart laterality disorders are dextrocardia (or *situs inversus* of the heart), which is a complete reversal of organ positioning, and heterotaxia of the heart, which represents any other lateral deviation from normal *situs.* Dextrocardia, often associated with reversed cardiac c-looping, represents the most common laterality disorder—almost half of all congenital laterality disorders.^23^ Importantly, aberrant LR asymmetry of the heart has been associated with morphological and functional defects. Cases of dextrocardia are often associated with double-outlet right ventricles, atrial septal defects, ventricular hypertrophy, pulmonary vein anomalies, visceral LR asymmetry, and, in rare cases, intracardiac anomalies and single ventricle formation.^7,8,23^ In addition, heart asymmetry is also often linked to aortic defects as detailed in the following sections. The overall prognosis depends on the presence of the accompanying conditions.^7^ It is widely accepted that the determination and control of cardiac LR asymmetry, like that of other organs, is a multifactorial phenomenon, driven by an array of temporally and spatially controlled molecular and biophysical signals.^4,24–26^

Recent evidence suggests that the heart looping directionality is independent of the early symmetry breaking of the overall body plan. For the latter, the molecular signaling has been studied in detail and has been thoroughly reviewed previously.^27–33^ Cardiac c-looping is tissue-intrinsic and not dependent on the well-studied LR morphogen gradients. Nodal is often considered as the main LR signal, which is asymmetrically expressed on the left LPM in bilateral animals and activates Pitx2 on the left side of the embryo.^34^ While the LR asymmetric expression of Pitx2 is crucial for proper organ *situs* and heart development, evidence suggests that Pitx2 asymmetry cannot explain the asymmetric looping of the heart or the determination of its direction.^25,27,35^ The independence of cardiac looping from the LR signaling has given traction to the idea that looping directionality is an actomyosin dependent mechanical property intrinsic to the HT.^36,37^ This is not an entirely new idea in and of itself since chick HTs were first observed by Manning and McLachlan to maintain its looping property and directionality when excised from the embryo and cultured *in vitro* 30 years ago and even earlier by Butler.^38,39^ Likewise, no drastically LR asymmetric cell division, death, or cell shape was observed in the developing tube.^37,40–45^ The mild asymmetry observed in the incorporation of progenitor cells from the SHF to the left and right HTs is also not enough to drive asymmetric looping.^46,47^ Similarly, cardiac contractions and the presence of the cardiac jelly and endocardial tubes are also disposable.^40,48^ Thus, better models for the understanding of the biophysical processes involved in looping are crucial.

## VASCULAR LATERALITY

In humans, the aorta and branches form in the fourth week of fetal development, with six pairs of primitive aortic branches connecting the aortic sac and dorsal aortae.^49–51^ Later on, most of these six branches regress and contribute to the formation of other parts of the vascular system or completely disappear, while only the fourth and sixth branches persist to exist. The fourth branches give rise to the left and right aortic arches (RAAs) under normal conditions.^50–52^

In very rare cases when the laterality of the aorta development pattern is reversed or disrupted, the patient can have a mirror-imaged right aortic arch (RAA), resulting in a single arch crossing over the right bronchus to the right of the trachea.^49–51,53^ The RAA usually associates with other congenital cardiac malformations such as tetralogy of Fallot or truncus arteriosus.^53^ In cases even rarer than the RAA, when both fourth aortic branches persist, a patient can have two arches forming a ring structure surrounding the trachea—narrowing it. A double aortic arch is usually an independent laterality malformation without associated anomalies.^50,53^

Transposition of the great arteries (TGA) describes a condition in which the aorta and pulmonary artery switch positions, causing the aorta to be connected to the right atrium, while the pulmonary artery is connected to the left atrium.^54–56^ The occurrence of TGA will cause the blood circled back from the body to be directly pumped out from aorta, while the oxygen-rich blood will be sent back to the lungs from the pulmonary artery.

The asymmetric development of the vascular system, and in particular, the great arteries, is often considered to be highly dependent on the heart looping, as their proper functionalities are closely related to heart morphogenesis and laterality. Defects in heart laterality have often associated vascular malformations, including TGA and RAAs.^49–56^ As such, the signaling pathways or mechanisms, which affect cardiac asymmetry, are found to cause vascular asymmetry. It is interesting to note that perturbations in asymmetric Pitx2 activation do not cause a complete reversal of the aortic orientations, much like the heart-tube itself.^35,57,58^ These findings suggest that factors influencing asymmetric development of the heart and great arteries could be remarkably similar and that cardiac cell chirality may play essential roles in both processes.

## CHIRALITY: CONNECTION BETWEEN MOLECULAR AND TISSUE CHIRALITY?

Cell chirality is a recently discovered property of the cell concerning its polarity, motility, and even morphology. It manifests itself as biased cell shape, directional cell alignment, chiral collective cell migration, biased positioning of intracellular organelles, or directional cell rotation/motion.^13,14,16,19,59^ While the specific mechanisms that regulate cell chirality are only beginning to be understood, it is widely accepted that the actin-based cytoskeleton, which is heavily involved in determining cell morphology and motility, plays a crucial role in cell chirality.^60–64^ The microtubule network is shown to be mostly disposable, which is consistent with its more prominent role in molecular transport.^16^ How the molecular chirality of actin and related structures relates to the cellular phenotype is not very well understood. Subsequently, to quantify such a novel morphological property of cells and study its biomechanical and biochemical regulation, novel bioengineering techniques are being developed and implemented and have resulted in significant progress in recent years.^10,12–14,65–67^

**FIG. 1.**
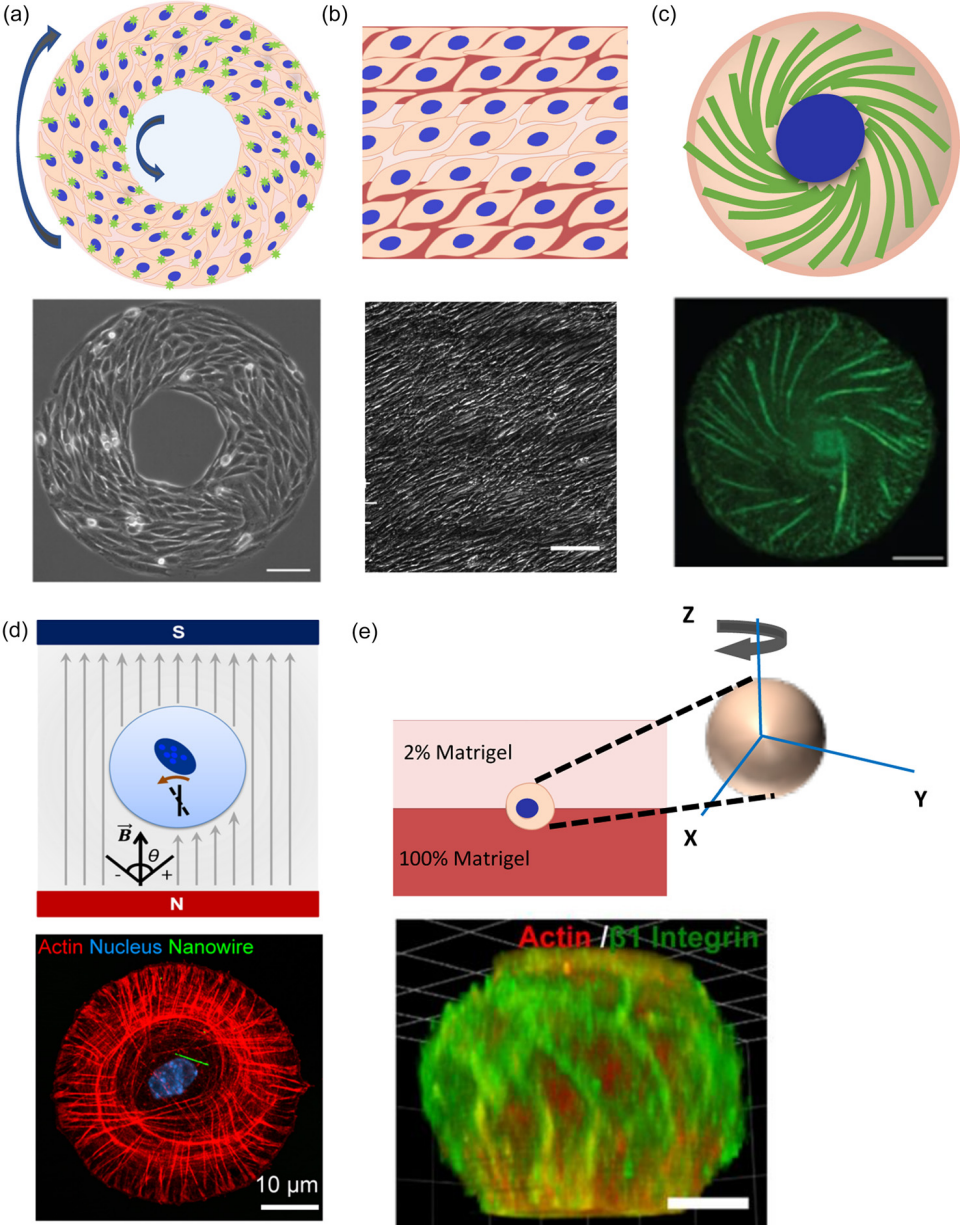
Some bioengineered methods for determining cell chirality. (a) Schematic of chiral cell alignment on ring-shaped micropatterns and centrosome polarization and migration (arrows) at pattern boundaries (top) with a representative phase contrast image (bottom). Reprinted with permission from Wan *et al.*, Proc. Natl. Acad. Sci. U. S. A. **108**(30), 12295–12300. Copyright 2011 National Academy of Sciences. The schematic was created using BioRender.com. (b) Schematic of chiral alignment of cells on alternating linear patterns of adhesive and non-adhesive substrates (top) and a representative phase contrast image (bottom) used reproduced with permission from Chen *et al.*, Biomaterials, **33**(35), 9019–9026. Copyright 2012 Elsevier. (c) Schematic (top) and fluorescent micrograph (bottom) showing chiral actin self-organization. Reprinted with permission from Tee *et al.*, Nat. Cell Biol. **17**(4), 445–457. Copyright 2015 Springer Nature Customer Service Center GmbH: Springer Nature. (d) Schematic (top) showing nano-wire based chiral torque determination by measuring the angle of nanowire rotation relative to the external magnetic field (gray arrows) and fluorescence image showing a nanowire and actin self-organization within a cell patterned on a circular protein island. Reprinted with permission from Liu *et al.*, ACS Nano **10**(8), 7409–7417. Copyright 2016 ACS. (e) Schematic (top) of the 3D bi-layer system with the cell sitting between two layers of Matrigel of different concentrations and 3D reconstruction (bottom) of fluorescently labeled spherical cell within the system. Reprinted with permission from Chin *et al.*, Proc. Natl. Acad. Sci. U. S. A. **115**(48), 12188–12193. Copyright 2018 National Academy of Science of the United States of America.

Micropatterning techniques have been particularly useful in the effort to quantify cell chirality. Mammalian cells cultured on ring-shaped micropatterns of fibronectin adopt a chiral alignment of cell boundaries relative to the circumferential direction of the pattern itself [Fig. 1(a)].^16^ The biased angles of cell alignment can be mathematically quantified, and the entire ring pattern can be statically defined as clockwise (CW) or counterclockwise (CCW).^16^ This bias is, in fact, specific to the phenotype of the cells, with myoblasts and cancer cells demonstrating a CCW bias, while endothelial, multipotent stem cells, and fibroblasts show a CW chirality.^16^ On the ring patterns, cell migration at the pattern boundaries is chiral as well. For example, cells with CW chiral bias move CW at the outer boundary and CCW at the inner boundary and vice versa for the CCW biased cells [Fig. 1(a)]. Patterned cells also demonstrate biases in the alignment of their nuclei and the direction of cell shape elongation.^64,65^ Similar nuclear alignment was also observed by Huang *et al.* using an outline etching image segmentation strategy.^60^ Interestingly, inhibition of actin polymerization led to the reversal of the CCW chirality of C2C12 mouse myoblasts, supporting the role of the actin cytoskeleton in chirality development.^16^ Similar biased alignment and migration were also observed with the vascular mesenchymal cells.^62^ Using line patterns of varying widths, we show that the large-scale chiral structure formation by epithelial cells requires cell–cell adhesions.^63^ Using alternating linear patterns of cell-adhesive fibronectin (FN) and non-adhesive polyethylene-glycol (PEG), Chen *et al.* similarly showed that vascular mesenchymal stem cells (VMSCs) obtain a biased alignment relative to the FN–PEG interface [Fig. 1(b)].^62,71^ Furthermore, they showed that with long-term culture, the cells migrate into the PEG layer and demonstrate biased migration along the axis of asymmetric cell alignment. This phenomenon is driven by cell polarization and actin stress fiber accumulation within the cells at the interface.^62^ They found that the chiral alignment of vascular mesenchymal cells is associated with actin stress fiber formation by showing that inhibition of actomyosin activities lead to the loss of chiral alignment. Likewise, several myosin isoforms have been identified that regulate LR asymmetry at various scales in *Drosophila*, including cellular chirality, in several tissues.^18,68–70^ Using linear patterns one-cell thick with a similarly narrow gap between patterns, Hu *et al.* demonstrated that the cells on neighboring patterns induce an asymmetric alignment after long-term culture.^72^ More recent attempts at quantifying this migratory bias using curved micro-stripes with pseudo-continuous CW and CCW curvatures showed no significant difference in migration velocity on CW vs CCW patterns.^73^ Finally, cell alignment and asymmetric migration of cells between alternating micropatterns were used to engineer custom myotubes to recapitulate *in vivo* muscle structures.^74^

To determine cell chirality *in situ*, i.e., without isolation of the cells from their natural environment or the need for micropatterning, inspired from early work done by Xu *et al.*,^75^ our lab has developed a new method to quantify cell chirality based on the positioning of the cell centroid with respect to the nucleus-centrosome axis, which is also commonly considered the front-back axis of cells.^12,76^ Most intriguingly, this approach can be applied to cells in non-patterned monolayers, as well as cells *in vivo*.^12^ This novel chiral marker has allowed us to associate cell chirality to tissue-level vascular endothelial permeability and chiral cell migration both *in vitro* and *in vivo*.^12,76^ The topic will be discussed in further detail in a later section.

The actin cytoskeletal dynamic is chiral. Micropatterning has allowed for the study of actin cytoskeletal chirality at a sub-cellular level, with exciting observations of biased rotation and tilting of the radial actin fibers in single fibroblasts or epithelial cells micropatterned on circular protein islands—using time-lapse fluorescence imaging to record the fluorescently labeled actin [Fig. 1(c)].^13,59^ A statistically significant bias in the direction of tilting has been observed, further establishing the role of actin dynamics in chiral morphogenesis. Additionally, the authors proved that the level of the cross-linking of radial actin fibers could regulate the directionality of actin swirling through genetic manipulation of α-actinin-1, an actin scaffold protein that crosslinks parallel actin filaments. Subsequently, the use of nanowires in conjunction with single-cell micropatterns was employed to quantify intracellular torque, presumably generated by the actin twirling in individual cells.^61^ The authors embedded ferromagnetic nanowires within the cytoplasm of individual cells micropatterned on circular protein islands and aligned them along an external magnetic field [Fig. 1(d)].^61^ They then measured the angle of the nanowire relative to the initial alignment along the magnetic field over time—which was then used to measure the intracellular chiral mechanical forces [Figs. 1(c) and 1(d)]. The authors found that chiral actin self-organization was accompanied by a chiral torque as well.^61^

While micropatterning represents a powerful tool for studying cell chirality, the 2D nature of micropatterned tissues may not accurately mimic *in vivo* tissue systems that exist as complex 3D structures. Recently, our group developed a 3D Matrigel bilayer system that allows for the quantification of chirality of single cells and multicellular cell spheroids derived thereof. The system is compatible with less adherent cell types, which cannot be micropatterned effectively [Fig. 1(e)].^14,20,21^ Cells are cultured on a layer of 100% Matrigel, allowed to attach, and then covered with a layer of diluted 2% Matrigel [Fig. 1(e)].^14^ Time-lapse images are then taken of the cells over several hours and analyzed to determine the rotation directionality [Fig. 1(e), inset].^14^ We observed chiral CCW rotation of Madin-Darby Canine Kidney (MDCK) epithelial cells and luminal spheroids derived thereof within the Matrigel bilayer. The bias is also consistent with chirality observed on 2D micropatterns. Overexpression of α-actinin-1-mediated actin cross-linking leads to reversal of cell chirality to a CW bias—which is also consistent with the reversed radial actin tilting of fibroblasts observed by Tee *et al.*^13,14^ Recently, a 3D hydrogel platform, in conjunction with Riesz transform-differential microscopy and computational kinematics, has revealed chiral neuronal growth cone motility as well, highlighting the ubiquity of cell chirality, even in organs not traditionally considered morphologically asymmetric.^77^ Similarly, the hydrogel–air interface has been used to demonstrate chirality in the growth patterns of neurites as they grow out of their hydrogel environment.^66^

We have further studied the phenotype-dependence of cell chirality using lineage-specific differentiation of human pluripotent stem cells (hPSCs). The hPSCs have no significant chirality within the Matrigel bilayer.^21^ However, they develop a chiral rotation upon differentiation, depending on the lineage and stage of differentiation. Mesoderm and cardiac lineages display a CW bias, whereas ectodermal and intermediate neural lineages and endoderm and intestinal lineages display a CCW bias.^21^ These data further suggest that chiral cell behavior is phenotype-specific and that it is determined upon differentiation to more terminal phenotypes during development. Interestingly, imposing chiral geometries has also been recently shown to regulate stem cell fate *in vitro*, suggesting that chiral mechanotransduction and differentiation may regulate each other.^75,76^ These findings have profound implications in the role of chirality in tissue morphogenesis and development.

Overall, bioengineering has been pivotal in studying cell chirality *in vitro* and *in vivo*, allowing for the development of chirality markers and implicating the roles of cell chirality in tissue-level asymmetric morphogenesis.

## CARDIAC LOOPING AND EMERGING ROLE OF CELL CHIRALITY

### Biomechanics of C-looping

Cardiac c-looping is a complex morphological event regulated both spatially and temporally.^24^ The process involves an LR symmetric bending of the linear HT, as well as an LR asymmetric torsion.^43^ This is shown by Voronov *et al.*, who used fluorescently labeled cells to mark the ventral most cells.^78^ They show that the cells that make up the outer concave surface of the looped heart are derived from the ventral most cells, suggesting that the ventral midline moves to the right side as a result of LR asymmetric torsion.^43^

Several models, reviewed thoroughly in the past,^24,25,43,79^ have been suggested to describe the origins and nature of this axial torsion, including LR differential growth, buckling of the growing HT within the cardiac cavity, oriented cell divisions, and asymmetric forces from the omphalomesenteric veins (OMVs) among others. Differential growth, oriented growth, and buckling could all physically lead to c-looping based on computational models, but they are not supported by *in vivo* evidence and also fail to describe how the directionality is determined.^25^ Similarly, while an LR asymmetric force from the OMVs would physically lead to LR asymmetric bending, mutated animal models with flipped OMVs maintained their looping directionality.^78^ Thus, these models suggest that ventral cell division leads to ventral bending but may fail to describe the origin and nature of the torsional force.

**FIG. 2.**
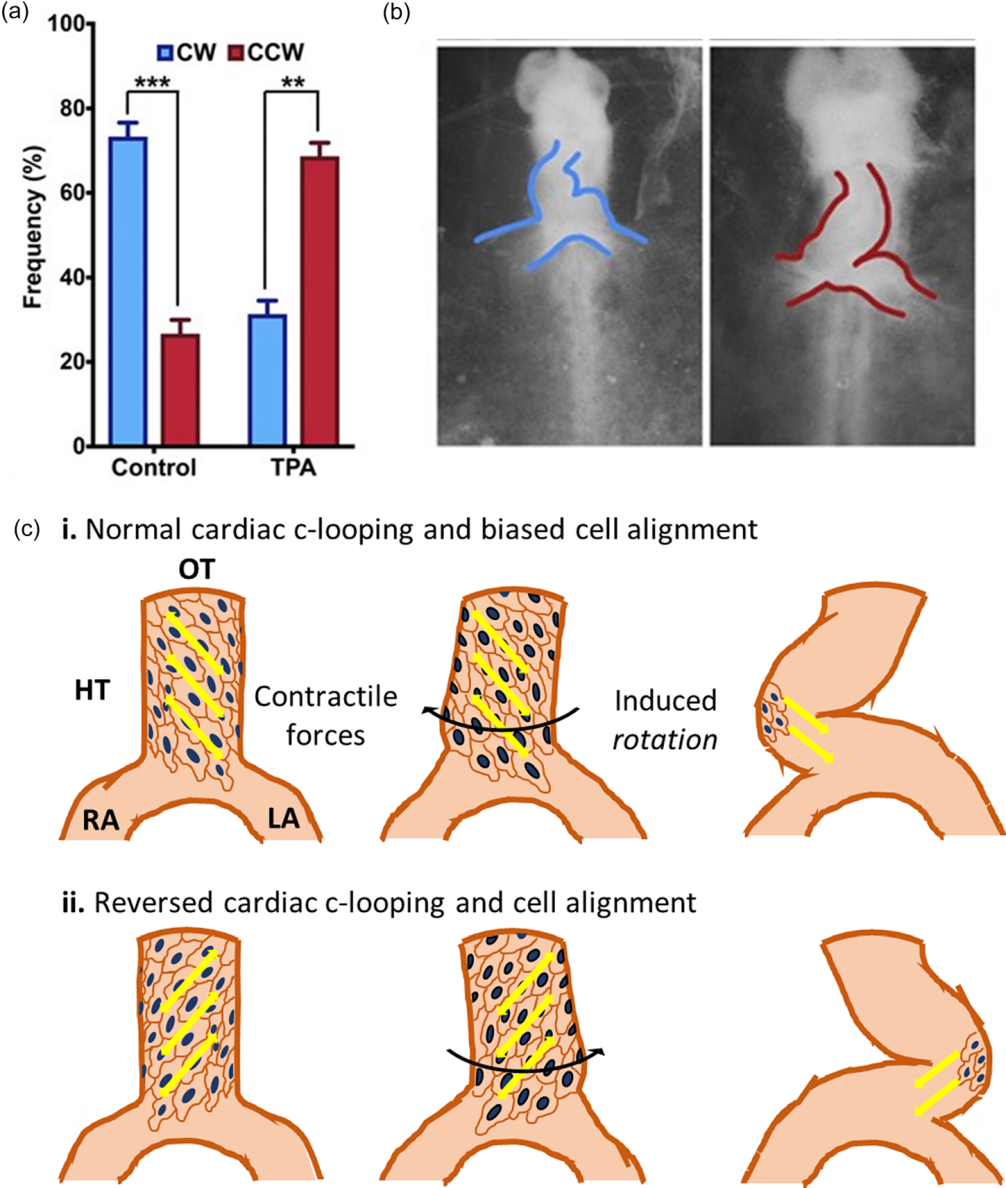
Cell chirality and cardiac c-looping. (a) Biases of cardiac cells isolated from the looping chick heart tube (HH9) show a CW bias in control cells and a reversed CCW bias in the cells treated with TPA (a potent activator of protein kinase C). (b) Phase contrast images of a wild-type (left) heart tube with correct looping directionality and a TPA-treated (right) heart tube with a reversed looping. Reproduced with permission from Ray *et al.*, Proc. Natl. Acad. Sci. U. S. A. **115**(50), E11568. Copyright 2018 Authors, licensed under a Creative Commons Attribution (CC BY-NC-ND) license.^20^ (c) Schematic showing normal (top) and reversed (bottom) chiral cardiomyocyte alignment, force generation, and looping in the looping heart tube.

This axial rotation was also described more recently by Le Gerrec *et al.* in mouse HTs.^46,80^ With 3D reconstructions of a mouse embryonic HT, they spatiotemporally define its shape and that of its surrounding tissue.^46^ They identify early LR asymmetries in the HT, including the rotation at the arterial port and subsequent asymmetry at the venous port. Likewise, Honda *et al.* used a computational cell-vertex based model to show that differential growth on the ventral side observed *in vivo* is sufficient to cause ventral bending, similar to finite element models published prior,^47,81^ but an LR asymmetric torsion was required to form the c-looped embryonic HT [Fig. 2(c)]. Interestingly, both models support the necessity of an initial torsional LR asymmetry to form the chiral helical shape of the looped HT, but the source of this asymmetry is still unclear.^47,81^

### Cell chirality drives cardiac morphogenesis

Several lines of evidence outlined thus far support the idea of cell chirality regulating tissue chirality. Such is especially true for tubular epithelial tissues, such as the gut and early heart-tube.^17,20,82^ Recent studies have established a direct link between the chiral cell shape and gut looping.^19,82^ These cells also have polarized actin and junctional proteins, with localization biased to the junctions aligned along with tissue alignment.^19^ These studies demonstrate that cells in the *Drosophila* hindgut adopt a chiral alignment, with the cells dominantly aligned to toward the left side of the embryo—a phenomenon that appears during the looping process but is lost once looping is complete.^18,19^ Similar observations were also made in the male *Drosophila* genitalia, which similarly loop and rotate during development.^18^ Similarly, Inaki *et al.* also demonstrate that these chiral cells also demonstrate chiral cell sliding.^82^ Our lab has since observed similar phenomena in the chick HT myocardium before and during c-looping, with an opposite LR alignment bias of cardiomyocytes toward the anterior-right direction, as well as N-cadherin and myosin-II polarization at the cell junctions aligned in the anterior right direction [Fig. 2(c)].^20^

Heart looping and cardiac cell chirality can be reversed by the same signaling. The studies in *Drosophila* have also revealed that chirality at the cell and tissue levels are reversed in *Myo31DF* mutants, an atypical myosin isoform, revealing a key regulator of chirality and chiral morphogenesis.^18^ No such regulatory mechanism had been found for vertebrate models until recently. Previously, we have shown that the CCW bias can be reversed upon perturbation of actin polymerization, but the CW biased cells remain unaffected.^16^ Our study in chick heart tube has revealed that the protein kinase C (PKC) signaling activation can reverse both cardiac cell chirality and chick heart looping *ex ovo* [Figs. 2(a) and 2(b)]. Recently, we found that activation of PKC signaling reverses the CW bias of endothelial cells as well.^76^ Also, PKC activation reverses cardiomyocyte alignment within the myocardium and actomyosin/N-cadherin polarization from the anterior right in wild-type HTs to the anterior left direction [Fig. 2(c)].^20^ As the directional localization of Myosin II and cadherin is associated with cellular contractility and mechanical tension, it is reasonable to speculate that the PKC regulates the looping through the anisotropy of mechanical forces. Furthermore, PKC is known to regulate the actin structure and associate with cardiac laterality diseases. Alterations in the actin structure may represent a target for PKC-mediated chirality reversal since PKC isoforms are known to regulate cardiac remodeling, but specific mechanisms involved in laterality determination are not known due to the ubiquitous nature of PKC signaling.^83,84^

An interesting observation in our study was that only the right side of the chick HT displays a strong biased Golgi polarization to the anterior-right direction, while the left myocardium has a slight bias along the posterior-left direction, suggesting that cardiac laterality may be derived from the right side of the heart.^20^ As expected, myocardial cells extracted from the right side of the chick myocardium at the looping stage display a CW bias, whereas those from the left side display a slight CCW bias.^20^ However, cells extracted from the entire heart tube as a whole still showed a significant, albeit a smaller, CW rotational bias.^20^ This finding is consistent with a biased PKC activation observed on the left side *in vivo*.^20^ These results show that while population biases are phenotype-specific, the spatiotemporal regulation of chiral biases is possible, opening up the possibility of exogenous control mechanisms that may enhance or perturb chiral morphogenesis in a multifactorial fashion.^26^ A recent study of the chick HT during looping confirmed the difference in the actin structure between the left and right myocardia, with the right side having longitudinally aligned actin fibers and the left myocardial actin aligned circumferentially.^85^ The authors further demonstrate that the actin-dependent directional cell rearrangement in the right myocardium primarily contributes to the LR asymmetric tissue deformation. This study further supports our finding that chiral looping is primarily derived from significantly chiral cells in the right HT and is driven by actin cytoskeletal dynamics.

## VASCULAR ASYMMETRY AND CELL CHIRALITY

While endothelial cell chirality does not seem to play a direct role in the development of vascular asymmetry, we have recently revealed a surprising role of cell chirality in vascular permeability and integrity, which affect the barrier function of the blood vessel.

### Vascular barrier

The important structure of the vascular networks, the vascular barriers, not only physically separate the interior environment from exterior tissues but also provide a bi-directional and selective transport of molecules.^86^ Endothelial cells, as the key cellular constituent of the barrier, wrap around the inner surface of vessels to form a tight layer that controls the infiltration of blood contents partly by regulating cell–cell junctions.^87^ The formation of a barrier is initiated by the contact of protruding lamellipodia between adjacent endothelial cells.^88^ Upon the retraction of lamellipodia, its mesh-like actin structure collapses into tightly bundled filopodia-like “bridges” that are connected by Vascular Endothelial (VE)-cadherin rich adherens junctions, and then these bridges are further matured with the formation of stress fibers and tight junctions.^88^ Under pathological conditions or when regulated by specific factors, the abnormal elevation of the permeability of the endothelial barrier is often associated with morphological changes of the actin cytoskeleton and junctional proteins along with the cell shape and polarity.^89,90^ For example, PKCs are essential regulators of the actin structure and vascular permeability, implicated in various vascular disorders such as inflammatory responses^89^ and diabetes mellitus.^90^ Together, these facts point to a potential association between actin-regulated junction formation and barrier functions, directly related to endothelial morphogenesis in angiogenesis and disease.

### Cell chirality regulates barrier function

**FIG. 3.**
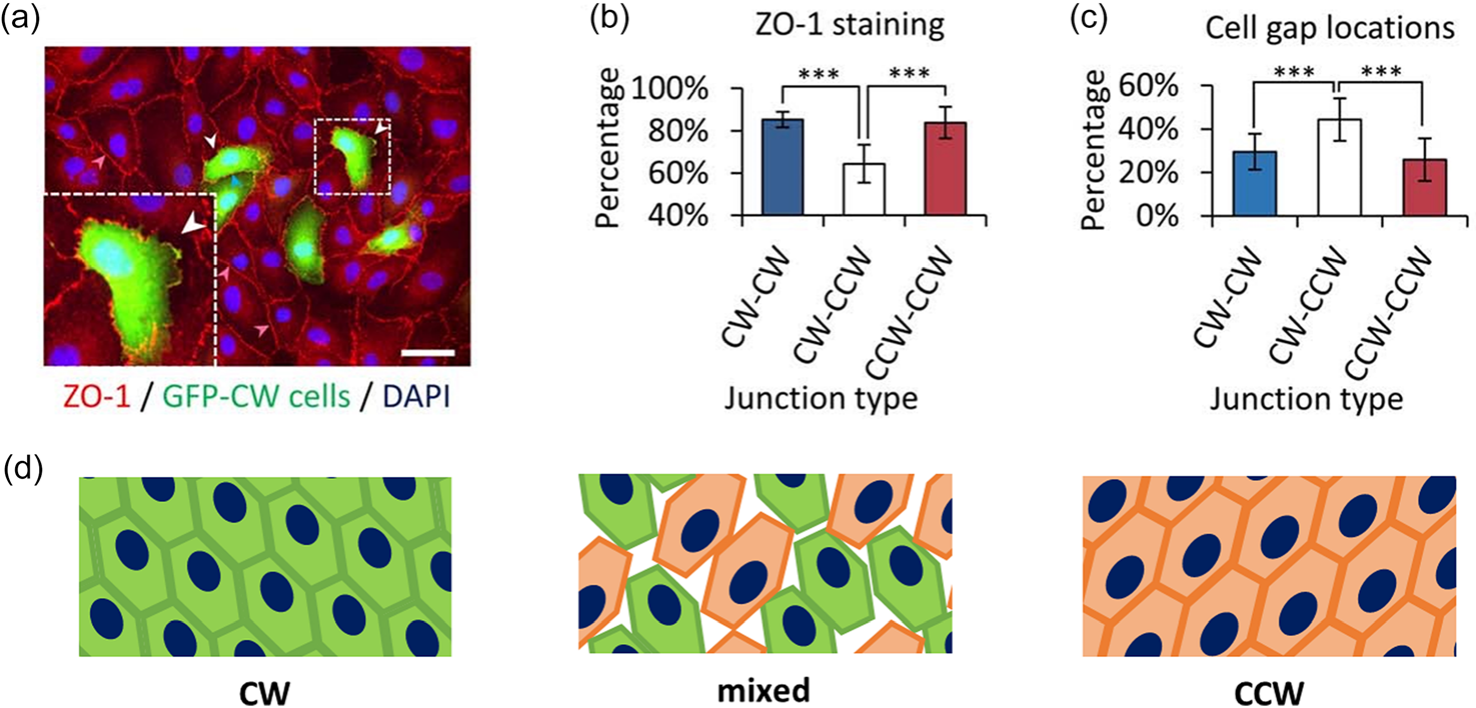
Cell chirality mismatch and cell-junction integrity. (a) Fluorescent images of CW cells (labeled green) in a mixture with unlabeled CCW cells. (b) ZO-1 staining shows a significantly higher intensity at CW–CW and CCW–CCW junctions than at mismatched junctions between the CW and CCW cells. (c) Quantification of the cell gap location shows that a significantly higher portion of cell gaps exists between the cells with mismatched chirality. (d) Schematic showing matched CW (left) and CCW (right) chiral cell monolayers with no gaps and mismatched chirality within a monolayer with intercellular gap formation and, therefore, impaired endothelial barrier integrity. Figure adapted with permission from Fan *et al.*, Sci. Adv. **4**(10), eaat2111. Copyright 2018 Authors, licensed under a Creative Commons Attribution (CC BY-NC) license.

Cell chirality, since arising from actin cytoskeleton,^10,13,16^ may be implicated in actin-regulated intercellular junction formations and vascular barrier functions. Along this direction, we have previously reported that the endothelial permeability can be regulated by cell chirality alternations.^76^ With PKC activation at a relatively low level via the treatment of small-molecule drugs, a dosage-dependent chirality shift of human umbilical vascular endothelial cells (hUVECs) from a CW bias to CCW bias was observed on ring-shaped micropatterns. This was associated with an interesting non-monotonic response in endothelial permeability, with a local peak at an intermediate level of PKC activation when the cells became most randomized in chiral biases. Further investigation on individual cell junction formation and chiral biases^12,76^ demonstrated the importance of uniformity in endothelial chiral biases for endothelial integrity [Fig. 3(d)]. Specifically, the cells with mismatched chirality have poorer junction formation than those with the same chirality (Fig. 3). However, how chirality regulates junction formation between cells is not well understood and further studies are required to determine biological mechanisms.

### Cell chirality in vascular barrier-related diseases

The mechanism of the endothelial cell chirality-regulated permeability change may also be implicative in vascular physiology and pathology. As stated above, PKCs are particularly important regulators for vascular functions: abnormally upregulated PKC activity is associated with various vascular disorders, and inhibition of PKC has been used as a treatment for some of these conditions. In diabetes mellitus, the increased formation of diacylglycerol (DAG) can lead to activation of PKC isoforms, which results in vascular defects, including abnormal upregulation of endothelial permeability and cytokine activation.^90^ Based on our findings, it is possible that the activation of PKC first alters the normal chiral bias of the vascular endothelial cells, leading to the randomization of the chiral morphology. The randomly biased endothelial cells, in turn, can disrupt cell–cell junction formation and eventually increase vascular permeability [Figs. 3(b) and 3(c)].

This role of cell chirality might also be implicated in vascular barrier dysfunction-associated neurodegenerative diseases such as Alzheimer's disease (AD). The breakdown of the blood–brain barrier (BBB) can occur in the early stage of AD pathogenesis, and certain AD-related proteins or risk factors have been shown to accelerate the BBB breakdown, leading to disruption in endothelial junctions and elevation in permeability.^91–93^ Similarly, these AD factors could potentially affect the chiral bias of the endothelial cells, which may eventually damage intercellular junctions and compromise the integrity of the BBB. To fully examine such possibilities, further investigations regarding the mechanisms of cell chirality in this direction will be necessary.

## PERSPECTIVES

While many questions regarding the role of cell chirality in cardiac c-looping remain unanswered, the field seems to be gathering around a loose consensus that widely accepts that asymmetry is an intrinsic property of the tissue at the time of looping. External LR signaling cues such as Nodal and Pitx2 derived from the dorsal mesentery, buckling forces from the anterior and posterior ends of the HT, HT elongation, cell addition from the SHF, planar cell polarization of myocardial cells, and LR asymmetric heart jogging make the process more robust—leading back to the idea of multifactorial and redundant determination of heart laterality.^28,34,94^ Increasing data suggest that intrinsic cell chirality can be spatiotemporally regulated and lead to the LR differences in cell alignment and cytoskeletal polarization observed within the HT.^20,21^ Supporting this even further are recent discoveries of tissue-intrinsic, right-specific, and Nodal-independent signaling pathways shown to regulate looping directionality.^37,38^ These new findings establish cell chirality as a novel candidate as the source of chiral axial torsion. Future work will likely involve the characterization of the physical nature of chirality, including chiral force generation. Additionally, there remains much potential in identifying the specific cytoskeletal proteins involved in the generation and regulation of these forces, including the role of PKC activation and actin polymerization/cross-linking. Computational models represent an exciting tool for rapidly testing various mechanical hypotheses and are likely to prove crucial in informing *in vitro* and *in vivo* studies.

While the asymmetry of vascular systems may associate with the cardiac c-looping, the role of cell chirality in regulating endothelial permeability is unique—establishing chiral cellular morphogenesis as a novel mechanism of regulating intercellular junction formation and affecting endothelium integrity.^76^ These findings reveal cell chirality as a novel regulator of vascular function with potentially broad implications in vascular disorders. Future works along this line might involve investigation of the specific mechanism of cell chirality regulating intercellular junction formations: in particular, how actin morphologies in oppositely biased cells differ from each other and how chirality mismatch disrupts the formation of intercellular junctions. Finally, the role of cell chirality in vascular barrier-related disorders is also worth investigating for the development of novel treatments.

Overall, cell chirality has demonstrated increasing importance in cardiovascular systems. The complete understanding of cell chirality in biological systems requires interdisciplinary collaborative effort from several fields of bioengineering, cell biology, and developmental biology.

## AUTHORS' CONTRIBUTIONS

T.R. wrote sections on cardiac asymmetry. H.Z. primarily wrote sections on vascular asymmetry. T.R., H.Z., and J.F. prepared the figures. J.F. edited the manuscript. L.Q.W. outlined and edited the entire review article. All authors gave final approval for publication.

## Data Availability

Data sharing is not applicable to this article as no new data were created or analyzed in this study.
